# Diverse high-risk B2 and D *Escherichia coli* clones depicted by Fourier Transform Infrared Spectroscopy

**DOI:** 10.1038/srep03278

**Published:** 2013-11-20

**Authors:** Clara Sousa, Ângela Novais, Ana Magalhães, João Lopes, Luísa Peixe

**Affiliations:** 1 REQUIMTE, Laboratório de Microbiologia, Departamento de Ciências Biológicas, Faculdade de Farmácia, Universidade do Porto, Rua de Jorge Viterbo Ferreira, 228, 4050-313 Porto, Portugal; 2 REQUIMTE, Laboratório de Química Aplicada, Departamento de Ciências Químicas, Faculdade de Farmácia, Universidade do Porto, Rua de Jorge Viterbo Ferreira, 228, 4050-313 Porto, Portugal

## Abstract

We aimed to develop a reliable method based on Fourier transform infrared spectroscopy with attenuated total reflectance (FTIR-ATR) to discriminate *Escherichia coli* clones from B2(n = 9) and D(n = 13) phylogenetic groups. Eighty-eight *E. coli* isolates belonging to phylogenetic groups B2(n = 39) and D(n = 49), including particularly widespread high risk clones or clonal complexes (HiRCC) ST131, ST69, ST393 and ST405 were studied. Spectra were analysed by unsupervised (hierarchical cluster analysis-HCA) and supervised methods (soft independent modelling of class analogy-SIMCA and partial least square discriminant analysis-PLSDA). B2-ST131 isolates were discriminated from B2 non-ST131 and D phylogroup isolates (ST69, ST393, ST405) by HCA, SIMCA and PLSDA. D-ST69, D-ST393 and D-ST405 isolates were also distinguished from each other and from other STs from phylogroup D by the three methods. We demonstrate that FTIR-ATR coupled with chemometrics is a reliable and alternative method to accurately discriminate particular *E. coli* clones. Its validation towards an application at a routine basis could revolutionize high-throughput bacterial typing.

The global dissemination in different settings of antibiotic resistant high-risk *Escherichia coli* clones or clonal complexes (HiRCC) with high virulence potential constitutes one of the major current challenges in clinical microbiology^1^,^2^. Particular *E. coli* clones from phylogenetic groups B2 (ST131) and D (ST69, ST393, ST405) with enhanced ability to colonize, persist and adapt to different hosts are recognized as extraintestinal pathogenic *E. coli* (ExPEC) lineages, which have largely contributed to the dissemination of β-lactam resistance determinants (mainly extended-spectrum β-lactamases and/or carbapenemases) in different countries^3^,^4^. Assessing the prevalence and dynamics of ST131 and other HiRCC by quick methods in the clinical setting would have a significant value for clinical, infection control and epidemiological purposes^2^.

Pulsed-field gel electrophoresis (PFGE) and multilocus sequence typing (MLST) have been useful for identification and discrimination of these *E. coli* clones at both local and global levels^3^,^4^,^5^, and although other alternative genotyping methods such as multilocus variable number of tandem repeats analysis (MLVA), allele-specific^6^,^7^,^8^,^9^ or real-time PCR^10^ or two-locus clonal typing have recently been proposed^11^, these methods are still time-consuming, laborious and/or expensive. Spectroscopic techniques such as Fourier transform infrared spectroscopy (FTIR) coupled with chemometric tools have demonstrated an interesting potential for the identification and typing of pathogenic and/or antibiotic resistant Gram positive and Gram negative bacteria at different taxonomic levels (species, subspecies, serotype and more recently at the strain level)^12^,^13^,^14^,^15^,^16^,^17^,^18^.

The basis of FTIR spectroscopy is the interaction of infrared radiation with a sample, in our specific case with the bacterial isolate, providing a specific fingerprint that reflects the structure and composition of the whole cell^19^. In the ATR mode, the infrared beam contact with the bacterial isolate and became attenuated. The magnitude of the attenuation depends on the bacteria in contact with the beam. The main advantages of FTIR-ATR spectroscopy are rapidity and reduced cost. This methodology requires no reagents or only low amounts of consumables, is non-destructive and environmentally friendly^20^.

In this study, we demonstrate the suitability of FTIR-ATR spectroscopy as a reliable alternative to discriminate diverse *E. coli* clones belonging to phylogenetic groups B2 (n = 9) and D (n = 13), including the particularly widespread ST131, ST69, ST393 and ST405 HiRCC.

## Results

### Isolates discrimination work-flow

The rationale of this study was firstly to discriminate the HiRCC B2-ST131 from the phylogenetic group D isolates. After, isolates from phylogroup B2 belonging to diverse STs were included to test FTIR-ATR ability in the discrimination of the ST131 from other B2 phylogroup isolates. Lastly, the discrimination within phylogenetic group D was evaluated. For this, in a first approach, the HiRCC ST69, ST393 and ST405 were discriminated from each other. Subsequently, it was evaluated the ability of FTIR-ATR to discriminate these HiRCC clones from other diverse STs of the phylogroup D.

### Spectra overview

FTIR-ATR spectra of all *E. coli* isolates tested revealed high similarity and bands associated with bacterial components such as lipids (3000–2800 cm^−1^), proteins/amides I and II (1700–1500 cm^−1^), phospholipids/DNA/RNA (1500–1185 cm^−1^), polysaccharides (1185–900 cm^−1^) and the fingerprint region (900–600 cm^−1^) were observed^19^. The main spectral differences were detected in the phospholipids/DNA/RNA and the polysaccharides regions (1500–900 cm^−1^), which were subsequently chosen for further comparisons. These regions have previously been used in other studies for discrimination at different taxonomic levels (including clones) in other bacterial species^16^,^18^,^21^.

### Discrimination of B2-ST131 isolates

**Figure 1:**
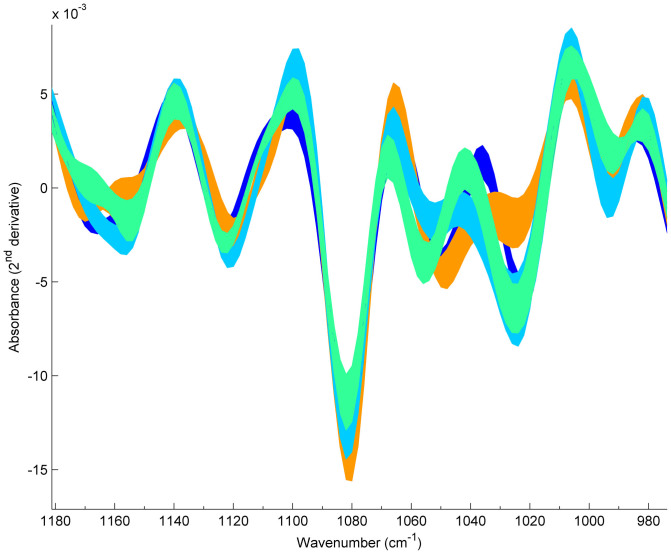
*Escherichia coli* FTIR-ATR spectra processed with SNV and Savitzky-Golay (9 points filter size, 2^nd^ degree polynomial, 2^nd^ derivative) corresponding to the mean ± one standard deviations in the region 1180–980 cm^−1^. Legend: 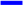 ST131, 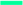 ST69, 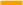 ST393 and 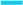 ST405.

**Figure 2:**
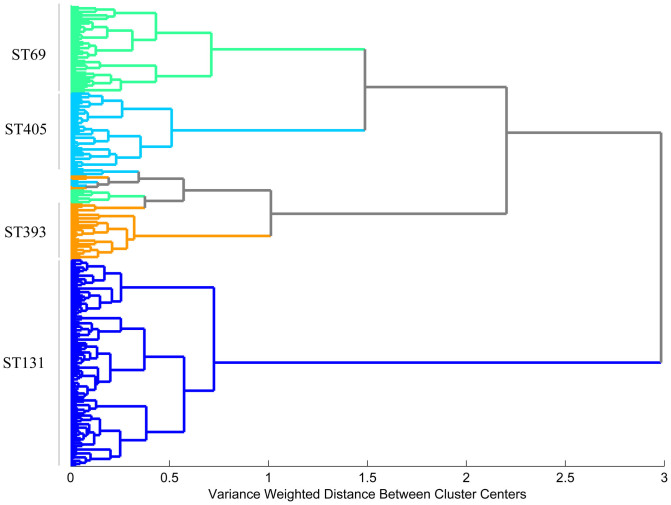
Dendrogram obtained from the 1500–900 cm^−1^ spectral region using the Ward's algorithm and 9 principal components (PCs) distance for isolates of the B2 (ST131) and D (ST69, ST393 and ST405) phylogenetic groups.

**Figure 3:**
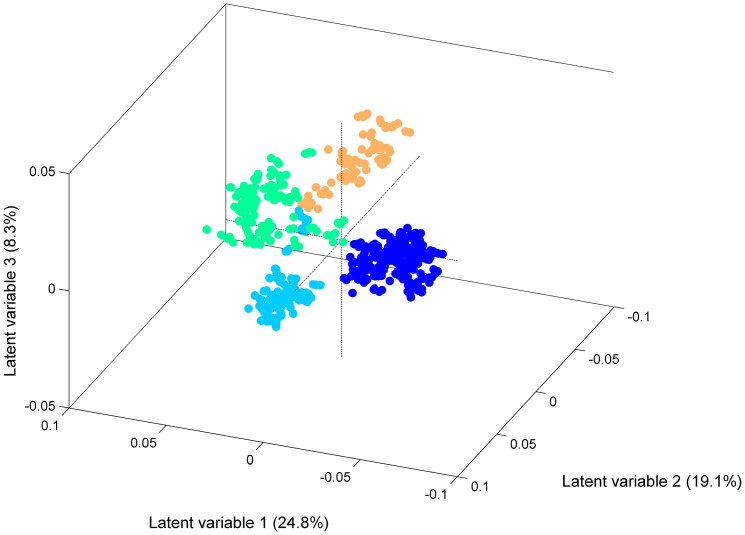
Score plot corresponding to the three first LVs of the PLSDA regression model with isolates belonging to phylogenetic groups B2 and D Legend: 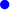 B2-ST131, 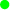 D-ST69 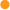 D-ST393 and 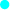 D-ST405.

B2-ST131 isolates were clearly discriminated from those belonging to phylogroup D by both HCA and PLSDA chemometric methods. The main spectral differences were observed in the regions of 1165–1155 cm^−1^, 1115 cm^−1^ and 1050–1030 cm^−1^ (attributed to aromatic vibrations, RNA ribose C-O stretching and diverse carbohydrates vibrations, respectively) (Figure 1)^22^. The dendrogram generated by HCA grouped all B2-ST131 isolates in one branch (including the single locus variant (SLV) ST1035) and all D *E. coli* clones in another branch containing ST69, ST393 and ST405 isolates (Figure 2). Consistently, the score plot obtained by PLSDA revealed the discrimination of B2-ST131 isolates by the first latent variable (LV1), which encompasses 24.8% of the total spectral variability (Figure 3). Both methods HCA and PLSDA, presented 100% sensitivity and 100% specificity in the discrimination of B2-ST131 meaning that all isolates of this ST were predicted as ST131 and all D phylogroup isolates were predicted as non B2-ST131 isolates. For sensitivity and specificity calculations the SLV isolates were excluded.

### Discrimination within phylogroup B2 isolates

**Figure 4:**
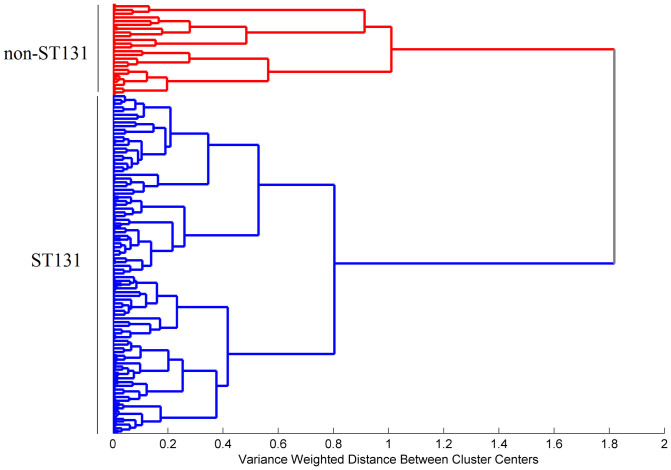
Dendrogram obtained from the 1500–900 cm^−1^ spectral region using the Ward's algorithm, the Mahalanobis distance and 10 principal components (PCs) for the isolates of the B2 phylogenetic group.

**Figure 5:**
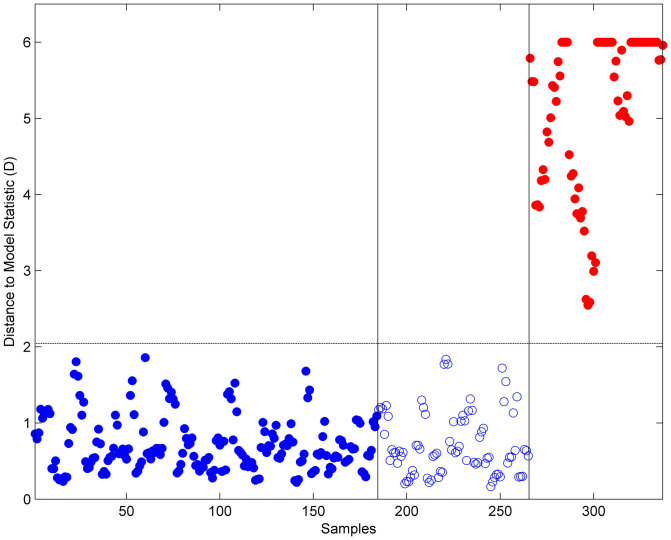
Distance to model statistics obtained by projecting ST131 and B2 non-ST131 samples on a SIMCA model calibrated with ST131 samples (note that values above 6 are truncated for better visualization). Legend: 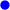 B2-ST131 and 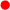 B2 non-ST131 (Unfilled circles indicated the samples used to test the model).

The comparison of FTIR-ATR spectra corresponding to ST131 isolates with those obtained from other B2 *E. coli* clones revealed that ST131 isolates were clearly discriminated from B2 non-ST131 isolates. The dendrogram obtained by HCA evidenced two clusters, one including all ST131 isolates and the SLV ST1035 and the other containing all non-ST131 isolates (Figure 4). A dendrogram generated only with ST131 isolates in the same conditions grouped isolates in two main clusters (data not shown) non-homologous to those observed by genotypic methods^23^. In addition, we performed a SIMCA model with ST131 isolates in order to test if the non-ST131 isolates were correctly predicted as not belonging to the modelled ST131 class. The correct discrimination of all the available samples (test samples and the non-ST131 isolates) was achieved with a three component model (Figure 5). All ST131 test samples appeared below the confidence limit confirming their assignment to the B2-ST131 class and B2 non-ST131 isolates appeared above the confidence limit meaning that they do not belong to that class. Both methods, HCA and SIMCA, presented 100% of sensitivity and specificity in the discrimination of B2-ST131 from B2 non-ST131.

### Discrimination within phylogroup D isolates

**Figure 6:**
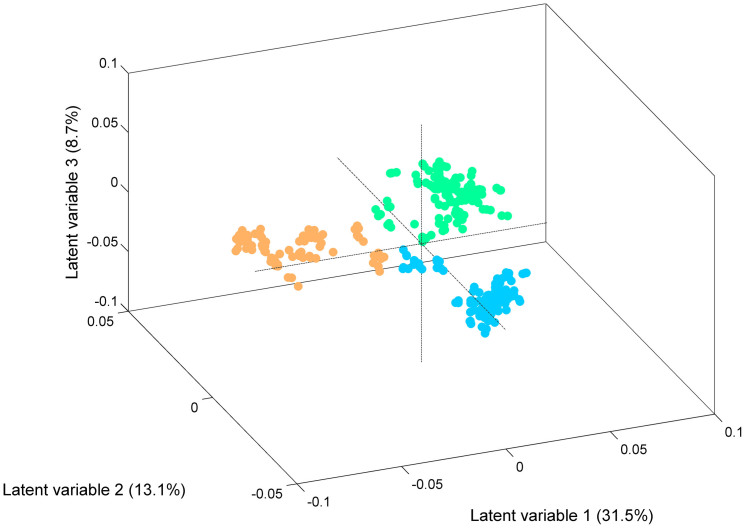
Score plot corresponding to the three first LVs of the PLSDA regression model with isolates belonging to phylogenetic group D. Legend: 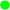 ST69 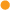 ST393 and 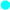 ST405.

The comparison of FTIR-ATR spectra of ST69, ST393 and ST405 isolates showed remarkable differences between ST69 and ST405 at 1025 cm^−1^ (associated with the S = O stretching of organic sulfoxides) and between ST393 and ST405 at 985 cm^−1^ (corresponding to the asymmetric stretching of (CH_3_)_3_N^+^) (Figure 1)^22^. The dendrogram generated by HCA with these isolates revealed that they were grouped in three clusters, containing respectively most ST69 (n = 11/13), all ST393 (n = 13) and most ST405 (n = 9/11) isolates (Figure 2). In this analysis, we obtained good sensitivity and specificity values for ST69 (85% vs 100%), ST393 (100% vs 83%) and ST405 (80% vs 100%). However, these clones could be perfectly discriminated by PLSDA (100% of sensitivity and 100% specificity). In fact, three clusters are evidenced in the score map, each one containing isolates from a given ST (ST69, ST393 and ST405), including the respective SLVs (Figure 6).

**Figure 7:**
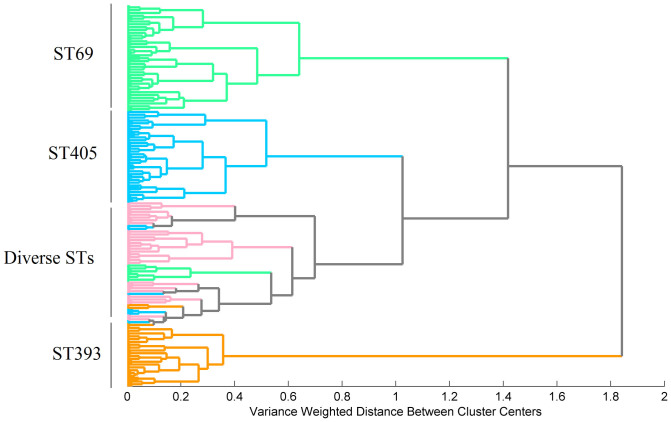
Dendrogram obtained from the 1500–900 cm^−1^ spectral region using the Ward's algorithm and 17 PCs for the isolates of the phylogroup D.

In addition, we tested the ability of FTIR-ATR to differentiate isolates belonging to ST69, ST393 and ST405 from isolates belonging to other diverse STs of the phylogroup D (hereby designated as other STD). The dendrogram generated by HCA showed that isolates grouped in four different clusters, one of them including the other STD isolates (n = 14) (Figure 7). HCA's sensitivity and specificity for the discrimination of ST69, ST393 and ST405 from the other STD isolates was 85% and 100%, respectively. Despite the diversity of STs included in the other STD group, they probably clustered together due to a higher similarity within them than with ST69, ST393 and ST405 isolates. Nothing can be inferred about the relative similarity among the STs of the other STD once just few isolates of each ST was considered in the analysis.

**Figure 8:**
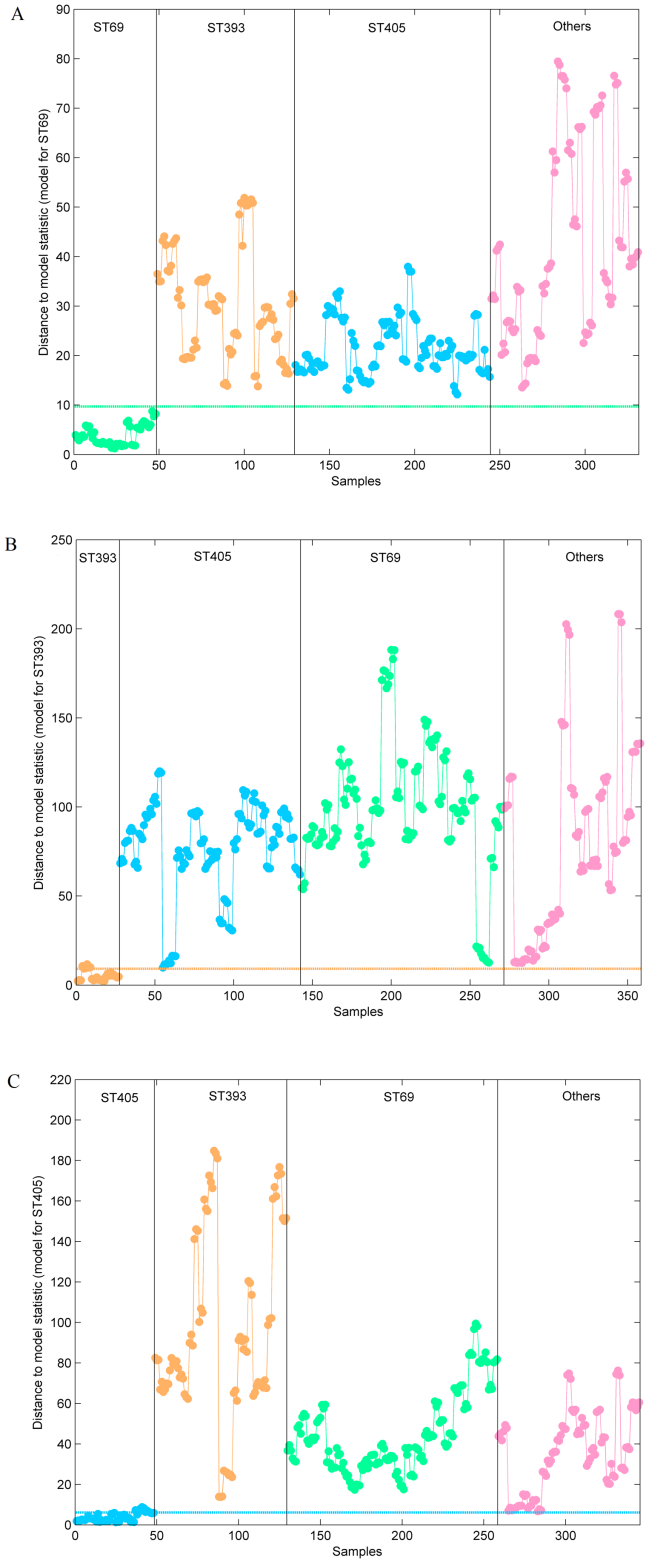
Distance to model statistics obtained for a SIMCA model for the three STs of phylogenetic group D: A) Model for ST69; B) model for ST393 and C) model for ST405. Legend: 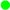 D-ST69 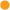 D-ST393 and 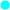 D-ST405 and 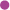 Diverse STs.

In order to test if all other STD isolates were correctly predicted as not belonging to ST69, ST393 and ST405, three independent SIMCA models were constructed for these three clonal groups. In each individual ST model, isolates from the remaining STs and from other STD were then projected into the model. All the test samples from ST69, ST393 and ST405 clonal groups were well assigned, whereas other STD isolates were in all cases predicted as not belonging to those groups (Figure 8) meaning that the SIMCA model had 100% of sensitivity and 100% specificity.

## Discussion

In this study, we demonstrate that FTIR-ATR spectroscopy coupled with chemometric tools is an alternative and reliable method to accurately discriminate particular *E. coli* clones belonging to B2 and D phylogenetic groups. The method proposed here consists on a reproducible framework where we discriminate sequentially: i) B2-ST131 from D-ST69, D-ST393 and D-ST405 *E. coli* clones; ii) B2-ST131 from other B2 *E. coli* clones; iii) ST69, ST393 and ST405; and iv) ST69, ST393 and ST405 from other D *E. coli* clones.

Strategies to shorten the time for the detection of these multidrug resistant and virulent clones and/or with potential application at a large-scale basis are being increasingly pursued^6^,^11^ since their application at a routine basis would have relevant clinical, infection control and epidemiological implications. Despite the increasing number of applications at different taxonomic levels, FTIR has very rarely been tested for clonal differentiation^16^,^17^,^21^. The study by AlRabiah et al. demonstrates the ability of FTIR to discriminate a few *E. coli* isolates involved in urinary tract infections including members of the ST131 clone. In our study, we included a larger and diverse sample of previously characterized *E. coli* isolates from different clones, demonstrating that FTIR might constitute a new and promising field in high-throughput bacterial typing. The results obtain herein proved FTIR precision (high consistency between biological and instrumental replicates) and accuracy (correct clonal group prediction).

FTIR provides a whole organism fingerprint^19^ that appears to be related with its phenotypic and genotypic features, since a good correlation was found between the assignments obtained by comparison of FTIR spectra and the STs determined by MLST, as observed previously in *Acinetobacter baumannii*^16^. FTIR seems to have a lower discriminatory power than MLST since SLVs of a given ST were not recognized, suggesting similarity of genotypic or phenotypic characters, as previously observed. However, isolates' clusterization did not correlate with that obtained by PFGE or with similarity of antibiotic resistance or virulence gene profiles^23^,^24^, suggesting that FTIR is possibly depicting more stable features. Its reliability for clonal discrimination will be further tested in a higher number of *E. coli* isolates and the possibility to extend the discriminatory power to other *E. coli* clones and eventually to other *E. coli* phylogenetic groups will be further explored.

FTIR spectrometers are available in many academic departments, laboratory research units or industries for a variety of purposes in chemistry and biochemistry such as characterization and quantification of chemical compounds or drugs, real time process monitoring or identification of potential bio threats or toxics^20^. The potential of this equipment for other goals may have been neglected over the years. We believe that if spectral acquiring conditions and the same equipment are assured, this method could be suitable for routine implementation in other laboratories enabling quickly and at a low cost the detection of high-risk *E. coli* clones, which would positively influence individual patient management decisions, infection control measures and monitorization of epidemiological trends. Finally, FTIR could be proposed as a reliable alternative to discriminate particular *E. coli* clones from B2 and D phylogenetic groups revolutionizing clinical bacteriology routines and high-throughput bacterial typing.

## Methods

### Bacterial strains

A set of eighty-eight *E. coli* isolates belonging to 22 clones from B2 (31 ST131, 1 ST1035, 1 ST12, 1 ST95, 1 ST126-like, 1 ST355-like, 1 ST799-like, 1 *fumC12*, 1 *fumC103*) and D (13 ST69, 10 ST393, 10 ST405, 1 ST2321, 1 ST964, 4 ST117, 1 newST, 1 ST648, 2 ST1011, 1 ST1325, 1 ST3177, 1 *fumC88*, 3 *fumC31*) phylogenetic groups were studied. They represent a diversity of previously characterized isolates identified in multiple countries, origins and periods (1980–2010), comprising diverse PFGE-types and variants (sharing identical virulence and/or antibiotic resistance profiles) from each clonal group^23^,^24^. Details about the bacterial isolates included in this study are summarized in Table 1.

### FTIR spectra acquisition

Spectra were acquired using a PerkinElmer Spectrum BX FTIR System spectrophotometer in the ATR mode with a PIKE Technologies Gladi ATR accessory from 4000–400 cm^−1^ and a resolution of 4 cm^−1^ and 32 scan co-additions. Isolates were grown on Mueller Hinton agar at 37°C for 18 h and colonies were directly applied in the ATR crystal and dried in a thin film. For each isolate, 9 spectra were acquired corresponding to three biological replicates (obtained from the same agar plate) and three instrumental replicates (obtained in three independent days).

### Spectral modeling

FTIR-ATR spectra were processed with standard normal variate (SNV)^26^ followed by the application of a Savitzky-Golay filter (9 smoothing points, 2^nd^ order polynomial and second derivative)^27^, mean-centred and analysed by unsupervised and supervised chemometric methods. All spectra (nine replicates for each isolate) was considered in the analysis and represented in the figures. The chemometric analysis were performed in Matlab version 6.5 release 13 (MathWorks, Natick, MA) and the PLS Toolbox version 3.5 for Matlab (Eigenvector Research, Manson, WA).

The unsupervised method employed was the hierarchical cluster analysis (HCA)^26^ using the Ward's algorithm to evaluate spectral similarity. Dendrograms produced by HCA were obtained after a principal component analysis (PCA)^26^, which ensured the robustness of the results. The supervised methods used were partial least square discriminant analysis (PLSDA)^28^,^29^ and soft independent modelling of class analogy (SIMCA)^30^. The PLSDA model is based on the PLS regression method^28^, and requires a previous knowledge about all the samples used. The model was calibrated considering all samples and the leave-one-sample-out cross-validation procedure in order to prevent overfitting^30^,^31^. The SIMCA model is based on the development of multiple PCA models, each one considering data for a particular class, and samples to be classified are then projected onto these models. In each model, 70% of randomly selected isolates of each ST were used for calibration (calibration samples) and 30% for testing (test samples). In our case, samples' class assignment^31^ was performed with the Euclidean distance. This model shows appropriateness when the objective is to classify samples within a defined set of classes and also to identify samples not belonging to any class.

## Author Contributions

C.S., Â.N. and L.P. participated in the conception and design of the study. C.S., Â.N. and A.M. contributed to data acquisition. C.S., Â.N. and J.L. performed data analysis. C.S., Â.N., J.L. and L.P. contributed to the preparation of the manuscript. All the authors read and approved the final manuscript.

## Figures and Tables

**Table 1:** Epidemiological details of the *E. coli* isolates used in this work. (H = Hospitalized patients; C = Community patients; F = Healthy volunteers; A = animals; E = Environment; S = Ready-to-eat salads)

Phylogroup	ST/*fumC*	Continent (N° countries)	N° of Isolates	Date	Origin^a^ (N° Isolates)	Ref
B2	131	Europe (7)	26	1991–2010	H (19), C (1), F (4), E (2)	[23]
	131	North America (1)	3	2008	A (3)	[23]
	131	Asia (1)	2	2006–2007	C (2)	[23]
	1035	Europe (1)	1	2005	H (1)	[23]
	95	South America (1)	1	2011	H (1)	*
	12	Europe (1)	1	2007	H (1)	*
	355-like	Europe (1)	1	2007	H (1)	*
	126-like	Europe (1)	1	2007	A (1)	[25]
	799-like	Europe (1)	1	2006/07	A (1)	[25]
	*fumC11*	Europe (1)	1	2010	H (1)	*
	*fumC103*	Europe (1)	1	2006	H (1)	*
D	69	Europe (3)	7	2002–2010	H (3), S (2), C (1), A (1)	[24]
	69	North America (1)	5	1999–2000	H (5)	[24]
	69	South America (1)	1	-	H (1)	[24]
	393	Europe (3)	5	2002–2006	F (3), C (1), H (1)	[24]
	393	North America (1)	3	1980–1999	H (3)	[24]
	393	Asia (1)	2	2006–7	C (2)	[24]
	2321	Europe (1)	1	2008	H (1)	[24]
	405	Europe (4)	9	2000–2008	H (7), C (2)	[24]
	405	Asia (1)	1	2004	H (1)	[24]
	964	Europe (1)	1	2003	H (1)	[24]
	117	Europe (1)	4	2006–2010	H (4)	*
	New ST^a^	Europe (1)	1	2010	H (1)	*
	648	Europe (1)	1	2006	H (1)	*
	1011	Europe (1)	2	2010	H (2)	*
	1325	Europe (1)	1	2010	H (1)	*
	3177	Europe (1)	1	2007	H (1)	*
	*fumC88*	Europe (1)	1	2010	H (1)	*
	*fumC31*	Europe (1)	3	2010	H (3)	*

^a^Double locus variant (DLV) of ST117;

*unpublished data.
